# Editorial: Representation in neuroscience and humanities

**DOI:** 10.3389/fnint.2022.1035367

**Published:** 2022-09-21

**Authors:** Marco Iacoboni, Deborah Jenson, Julie Uchitel, Leonard E. White

**Affiliations:** ^1^Department of Psychiatry and Biobehavioral Sciences, David Geffen School of Medicine, Neuromodulation Lab at the Ahmanson-Lovelace Brain Mapping Center, University of California, Los Angeles, Los Angeles, CA, United States; ^2^Romance Studies, Trinity College of Arts & Sciences, Duke Global Health Institute, Duke Institute for Brain Sciences, Duke University, Durham, NC, United States; ^3^Department of Paediatrics, University of Cambridge, Cambridge, United Kingdom; ^4^Department of Neurology, Duke University School of Medicine, Duke Institute for Brain Sciences, Duke University, Durham, NC, United States

**Keywords:** representation, neuroscience, humanities, perception, neurohumanities

Both neuroscience and the humanities seek to understand the nature of representation and simulation, yet seldom in dialogue or collaboration. Neuroscience tries to assign metrics of brain activity to specific representations of the outer world. For instance, a flower can be represented by population level activity in the ventral visual stream, or in terms of the motor plans required to grasp it in parietal, premotor and motor areas. It is also represented in much richer associations spanning activity and computations across systems and networks. The humanities encompass what ancient Greeks named *mimesis*, a complex field ranging from representations of internal and external worlds in arts and literature, to social synergies and “contagion,” to the aesthetic *gestält* of cultures. Natural sciences and engineering use the related interdisciplinary concept of biomimesis. Both neuroscience and humanities have critiqued the notion of representation as mental simulation of objective reality in the individual brain, in favor of non-solipsistic, multidimensional, and dynamic frameworks, including paradigms of distributed mind and 4E (embodied, embedded, enacted, and extended) cognition.

Scaling issues complicate interdisciplinary efforts to theorize representation. In neuroscience, the scale might be at the level of the neural coding of a transient event (a briefly flashed stimulus in a laboratory experiment), or of carefully controlled events unfolding over longer time scales (a conversation, a film). In literature, even a single metaphor, such as Proust's madeleine in *In Search of Lost Time*, is prized for its introduction of a potentially infinite wealth of associations and mental states, beyond quantifiable data.

Research into representation benefits from dialogue and collaboration between the neurosciences and the humanities, through the construction of conceptual and/or experimental bridges. This Research Topic encourages neuroscientists to engage with mimesis, and humanists to engage with systems neuroscience as a portal toward a biological or phenomenological understanding of representation in embodied, experiential, and social senses. Both neuroscientists and scholars in the humanities may ask: How do neurobiological conceptions shed light on representation in mimetic terms? How do neurosciences and humanities visualize or describe representation itself, as a dimension of mental, embodied, and social functioning? Knowing that representation of the thoughts and intentions of others may be grounded in the sensorimotor transformations of distributed circuits in the brain, what new questions can we ask about mimesis? How does network neuroscience account for that? How might brain machine interface shape humans' representation of the internal and external world? These questions open a new frontier in integrative neuroscience, a neurohumanities frontier that calls for integration, or mediation, of the methods, assumptions, and practices. Bridging these two fields, which may initially appear as extremes, is achieved in this collection through questions aimed at intersubjective cognition, bilateral neuroscientific and humanistic analyses of texts and art, and neurocognitive computational models of representation.

Gambino and Pulvirenti present imagination as a neurocognitive framework for understanding embodied simulation. Embodied simulation, neuroscientifically understood through activation of sensorimotor mechanisms, may occur during reading in which the reader may cognitively “imitate” or perform “world construction” of the literary text they are immersed in. The authors argue that scientific research may benefit from literature's dynamic and embodied simulation-based interpretation of imagination when formulating questions about cognitive faculties such as perception, imitation, and simulation.

Embodied simulation is then explored by Agarwal, who presents an analysis of complementary and alternative medicine (CAM) providers' practices in their treatment of breast cancer survivors. In this original research study, recruited CAM providers undergo semi-structured interviewing that seeks to reveal how providers “tune in” to their patients' internal states and “co-create experiences of pain” through embodied simulation, imagination, and intentional vulnerability. The article then interprets the study's findings within the context of the mirror neuron system. It describes how mimetic self-reflexivity and intersubjectivity may inform research on self-recognition and self-other discrimination with implications for complementary healing practices among breast cancer survivors at risk of post-surgical pain.

Hipólito rejects the common analogy made between neurocognitive activity and computers, arguing that properties of the models used in computational cognitive neuroscience (i.e., information, representation) do not literally exist in the brain. The author presents Dynamic Causal Modeling (DCM) and Dynamic Systems Theory (DST) as alternative approaches for dynamically describing neurocognitive activity.

Tying together cognition, cultural transmission, and representation, Holhol et al. present the importance of ‘cognitive artifacts' as essential tools for cumulative culture and cultural innovation. According to the authors, cognitive artifacts are external artificial devices designed to serve a representative function, a simple example being a map, and more complex examples being literary oral formulae or mathematical proofs. Through three examples from cognitive history, namely, Homerian epics, Euclidean geometry, and Roman law, the authors describe how artifacts shape ‘cognitive niches' for innovation across historical timescales.

Reilly argues that neuroscientific visualizations of mental functioning such as Ramón y Cajal's pen and ink renderings of pyramidal neurons and glial cells fall within the mimetic tradition and bring non-realist techniques to bear in visualization of brain and mind. Once new methodological links between humanities and neuroscientific visualizations of mental functioning are forged, aesthetic works in the humanities such as Lucretia Martel's film “The Headless Woman” may shed new light on representational strategies in neuroscience to make otherwise invisible psychic phenomena observable.

Rodríguez Villar explores how structural representations of time in theater can be used as a lens for investigating human perception and cognition in the dramatic form of the *Autos sacramentales* by the early modern Spanish playwright Pedro Calderón de la Barca. The Christian understanding of time and the characters' perceptions of time presented in this text is linked to neuroscientific evidence of how the human brain processes time, such as circadian rhythms and hippocampal-driven memory encoding.

Representation in these six articles extends beyond the symbol processing that was long interpreted as an Archimedean point in neuroscience. They ask new questions about both science and arts as finely honed mimetic systems of embodied sense-making, overlapping with world-making. They reflect broad potential for integrated and intersectional methodologies bridging neuroscience and humanities.

## Author contributions

MI, DJ, and LEW functioned as editors of the associated Research Topic, Representation in neuroscience and humanities. JU assisted the editors. All authors contributed to the article and approved the submitted version.

## Conflict of interest

The authors declare that the research was conducted in the absence of any commercial or financial relationships that could be construed as a potential conflict of interest.

## Publisher's note

All claims expressed in this article are solely those of the authors and do not necessarily represent those of their affiliated organizations, or those of the publisher, the editors and the reviewers. Any product that may be evaluated in this article, or claim that may be made by its manufacturer, is not guaranteed or endorsed by the publisher.

